# The Diagnostic and Clinical Significance of TFE3 Immunohistochemical Nuclear Expression in Solitary Fibrous Tumour

**DOI:** 10.1155/2020/8232803

**Published:** 2020-05-28

**Authors:** Luting Zhou, Anran Wang, Guoli Yu, Jun Zhou, Haimin Xu, Chaofu Wang

**Affiliations:** ^1^Department of Pathology, Ruijin Hospital, Shanghai Jiaotong University School of Medicine, Shanghai, China; ^2^Department of Pathology, The 960th Hospital of PLA, Jinan, Shandong, China

## Abstract

The expression of TFE3 (transcription factor E3) in solitary fibrous tumours (SFTs) and their histologic mimickers was investigated, and the diagnostic value and clinical significance of TFE3 nuclear expression in SFTs were explored. Immunohistochemical analysis for TFE3 was performed on 50 cases of SFTs that were surgically resected. The controls were sample tissues from malignant peripheral nerve sheath tumour, synovial sarcoma, dedifferentiated liposarcoma, spindle cell lipoma, and dermatofibrosarcoma protuberans. The survival of patients with TFE3-positive and TFE3-negative expressions was assessed through the Kaplan-Meier analysis. In 44 of 50 (88%) SFTs, nuclear immunoreactivity for TFE3 was detected. The TFE3 expression was negative in all samples of synovial sarcoma, malignant peripheral nerve sheath tumour, dermatofibrosarcoma protuberans, and spindle cell lipoma and weakly positive in 2 of 10 cases of dedifferentiated liposarcoma. Fluorescence in situ hybridization (FISH) confirmed that the expression of the TFE3 protein is not caused by gene translocation. There was no statistical significance between the association of the TFE3 expression and SFT patient prognosis. Therefore, TFE3 is capable of enhancing the differential diagnosis of SFTs and their histologic mimickers and can be potentially used as a diagnostic marker. The findings also offer valuable insights into SFT diagnosis, aetiology, and associated molecular mechanisms.

## 1. Introduction

Solitary fibrous tumour (SFT) is a ubiquitous fibroblastic mesenchymal tumour, which has the potential to affect any region of the body. It was first described in the pleura by Klemperer and Rebin in 1931, and later in 1942, these tumours were also observed in multiple sites [1, 2]. Most SFTs are painless, present with a defined boundary, and slow growing. Malignant tumours often show local infiltration and their diameter ranges between 1 and 30 cm. The sections of tumours have been frequently observed having a whitish and firm appearance with multiple nodules. Malignant tumours are characterized by aggressiveness with haemorrhagic necrosis and cystic degeneration [3, 4]. SFTs are morphologically diverse, characterized by an alternate distribution of hypocellular and hypercellular areas, and haphazard growth pattern of spindle cells mixed with fibrous bundles and haemangiopericytoma-like vessels. According to the histomorphologic difference, SFTs can be classified into classical SFTs, giant cell SFTs, fat-forming SFTs, and malignant SFTs [5, 6]. The histological features of SFTs are different from spindle cell lipoma, dermatofibrosarcoma protuberans, synovial sarcoma, and malignant peripheral nerve sheath tumours.

The genetic mechanism of SFT activity has been studied and has received extensive attention clinically. In recurrent SFTs, a fusion gene of NAB2-STAT6 was detected by comprehensive sequencing [7]. STAT6 is a newly discovered member of the transcription factor family that can combine with the target gene regulatory region. It functions doubly as an activator of signal transduction and transcription. NAB2-STAT6 gene fusion is an important driving gene of SFT. Overexpression of NAB2-STA6 can induce cell proliferation and EGR gene expression. Therefore, nuclear STAT6 immunoreactivity using a STAT6 antibody is a highly sensitive and helpful marker of SFTs. Additionally, Vivero et al. found that the GRIA2 gene was highly expressed in SFT, and GRIA2 tested positive in 80% of SFTs [8]. Typical SFTs show a patternless architecture characterized by a combination of hypocellular and hypercellular areas separated by thick bands of collagen and thin-walled branching haemangiopericytoma-like vessels. While most cases of SFTs are benign, their behaviour is unpredictable. About 10% SFT cases behave aggressively. Malignant SFTs show an increased rate of mitosis (>4 mitoses per 10 HPF), cytological atypia, tumour necrosis, and infiltrative margins.

Transcription factor E3 (TFE3), also known as weight chain immunoglobulin enhancer 3, belongs to the MITF (microphthalmia-associated transcription factor) family, which also comprises transcription factor EB (TFEB), transcription factor EC (TFEC), and MITF [9–11]. The MITF family is an important regulator of cell differentiation and development and is involved in the regulation of tumorigenesis [12]. The *TFE3* gene is 14.78 kb long and is positioned in the short arm of X chromosome p11.22. The TFE3 protein is widely distributed in the human body and binds to the DNA in the form of a homologous dimer or a heterodimer to act as a transcription factor and play the regulatory role for multiple genes and signalling pathways [13]. Additionally, the TFE3 protein interacts with other regulators of transcription such as Smad3, E2F3, and LEF1 and plays a crucial role in the growth and expansion of cells and differentiation of osteoclasts and macrophages [14]. *TFE3* acts as an oncogene which is commonly rearranged in many tumour types, such as translocation of Xp11.2 leading to renal cell carcinoma, alveolar soft tissue sarcoma (ASPS), a subset of perivascular epithelioid cell tumours (PEComas), and partial epithelioid hemangiosarcoma [15, 16]. It has been observed that increased TFE3 protein expression is not associated with gene translocation in many tumours such as hepatic angiomyolipoma, granulosa cell tumours, and solid pseudopapilloma of the pancreas [17, 18].

A consistent finding in SFT biology has been that of the NAB2-STAT6 fusion, thus making STAT6 immunohistology reliable for detecting the fusion gene, although the molecular determinants in SFT malignancy still remain to be unravelled. TFE3 plays important roles in oncogenesis, although the link between TFE3 expression and SFTs has not yet been assessed. We hypothesized that TFE3 may be a useful diagnostic marker of SFT and, thus, sought to assess TFE3 expression in 50 SFTs via immunohistochemical analysis; compared these expression patterns to those of other tumour types to assess the specificity of TFE3 marker for SFTs; and evaluated the association of TFE3 with gender, tumour size, mitotic rate score, and SFT patient prognosis.

## 2. Materials and Methods

### 2.1. Patient Samples

A total of 50 surgically resected SFT specimens were acquired from the Pathology Department of the Ruijin Hospital in Shanghai, China. Samples were collected from August 2014 to May 2018. To assess the specificity of TFE3 to SFTs, we also carried out the tests on samples of malignant peripheral nerve sheath tumour, synovial sarcoma, dedifferentiated liposarcoma, spindle cell lipoma, and dermatofibrosarcoma protuberans. All specimens were fixed with 10% formaldehyde and embedded in paraffin. The cut sections (4 *μ*m thick) were then stained with haematoxylin and eosin.

### 2.2. Immunohistochemical Staining and Evaluation

A Dako Omnis automated staining platform was used for immunohistochemical staining of whole tissue sections (4 *μ*m thick) embedded in paraffin and formalin-fixed. For details regarding the antibodies used in this study, see Table 1. The antibodies were optimized using Ventana DAB detection kit (Ventana Medical Systems), and standard quality control procedures were carried out.

The immunohistochemical staining of TFE3 expressions was quantitatively scored on a scale of 0 to 3+ depending on the extent and the intensity of staining. The sample scored 0 if it did not stain at all; 1, if it stained weakly (light brown); 2, if it stained moderately intense (brown); and 3 in case of high intensity (dark brown). The percentage of TFE3-positive nuclei was scored as follows: 3 (>70%), 2 (40-70%), 1 (10-40%), and 0 (<10%). A histologic score was then generated by multiplying these two scores together, and the final positivity scores were assigned based on this histological score as follows: 3+ (a score of 9), 2+ (a score of 4 or 6), 1+ (a score of 2 or 3), and negative (a score of 0 or 1).

### 2.3. Fluorescence In Situ Hybridization (FISH) Assay

For the FISH assay, a Zytolight SPEC TFE3 Dual colour break-apart probe was used on 4 *μ*m thick formalin-fixed and paraffin-embedded tissue sections. The signals of FISH were evaluated using a microscope Olympus BX51TRF (Olympus, Japan) by applying a triple-pass filter (DAPI/Green/Orange; Vysis). Signals were deemed split when the distance between red and green signals was ≥2 signal diameters. The TFE3 cases were FISH positive when the tumour samples contained more than 15% split signals [19].

### 2.4. Statistical Analyses

The TFE3 expression was correlated with patient age and tumour size using the unpaired *t*-test. Likewise, the expression of TFE3 was related to gender and mitotic rate score using the Fisher exact test. The event-free survival was estimated using the Kaplan-Meier method. A *p* value < 0.05 was statistically significant.

## 3. Results

### 3.1. Clinicopathological Findings

We analysed a total of 50 SFT cases and 50 other lesion types as controls. A summary of the clinicopathological features is presented in Table 2. Among the 50 SFT patients, 23 (46%) were male and 27 (54%) were female; their ages are in the range of 18-82 years (mean age: 52.08 years). When the tumour localization was assessed, they occurred in the retroperitoneum (*n* = 14), pelvic cavity subperitoneal tissue (*n* = 9), pleura (*n* = 4), lung (*n* = 5), pancreas (*n* = 4), kidney (*n* = 2), parotid (*n* = 1), limbs (*n* = 2), buttocks (*n* = 3), central nervous system (*n* = 2), intestinal wall (*n* = 2), groin (*n* = 1), and bladder (*n* = 1). The dedifferentiated liposarcoma was found in the retroperitoneum, and the synovial sarcoma occurred mainly in the limbs.

**Figure 1:**
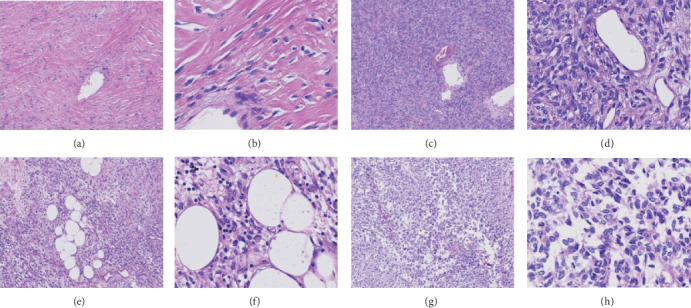
Microscopic evaluation for SFTs. Classic SFT with low-to-intermediate cellularity and densely collagenized, observed at (a) 100x and (b) 400x. Cellular SFT with ovoid-to-spindled cells in dense cellular proliferation arranged in a haphazard pattern in a less prominent stroma at (c) 100x and (d) 400x. Fat-forming SFTs with a variably prominent adipocytic component at (e) 100x and (f) 400x. Malignant SFTs with enhanced mitotic activity and significant cell atypia at (g) 100x and (h) 400x.

The diameters of the largest and the smallest tumours were 19 cm and 1 cm, respectively. On sectioning, most SFTs exhibited a multinodular, whitish, and firm appearance. A further morphologic examination confirmed the diagnosis of all SFT cases. Histologically, 26 cases were of classic SFTs. The tumours of SFT present as a well-circumscribed but nonencapsulated mass, and there were dense and sparse areas with no special regularity in the cellular arrangement. At high magnification, spindle, oval, and round cells with less cytoplasm were observed. The chromatin was homogeneous, the nucleolus was not obvious, the atypia was not obvious, and there was no pathological mitotic image. The lesion presented a pattern mainly of spindle cells in low-to-moderate cellularity scattered within a prominent matrix made of collagen. A total of 10 cases were determined as cellular SFT, whose predominant histologic pattern was of ovoid-to-spindled cells in a densely cellular proliferation arrayed in a haphazard pattern, and contained in a less prominent stroma. Three cases were of fat-forming SFTs; their predominant histologic pattern was a variably prominent adipocytic component; one case was of giant cell SFT with pseudovascular or sinusoidal lacunae and multinucleated giant cells; 10 cases showed enhanced mitotic activity (>4/10 HPF) with or without spontaneous coagulative necrosis and were diagnosed as malignant SFTs (Figure 1). Table 2 presents the clinicopathologic features in detail.

### 3.2. Immunohistochemical Findings

**Figure 2:**
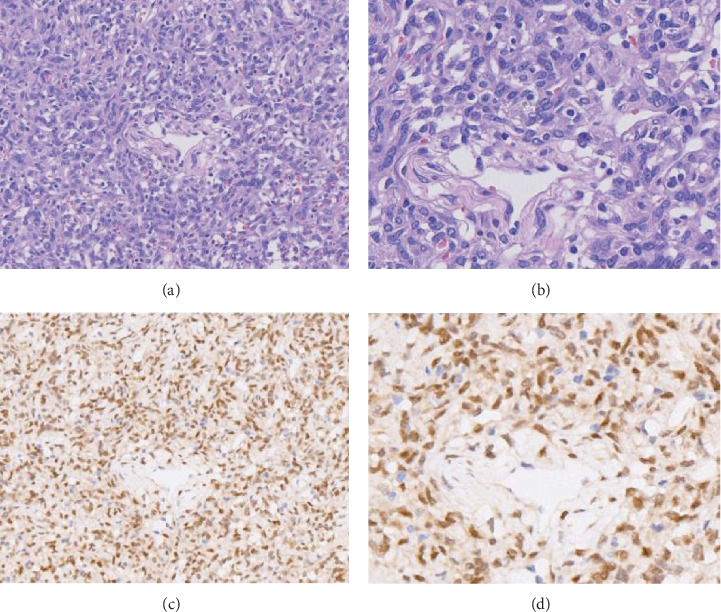
Histopathological characteristics of SFTs and the corresponding TFE3 immunohistochemical expression. Routine H&E staining of SFT with “staghorn” vasculature at (a)100x and (b) 400x. Robust TFE3 expression in the corresponding SFT as assessed by immunohistochemistry at (c) 100x and (d) 400x.

We conducted immunohistochemical staining for TFE3 in all 50 SFTs and observed malignant peripheral neurilemmoma (*n* = 10), synovial sarcoma (*n* = 10), spindle cell lipoma (*n* = 10), dermatofibrosarcoma protuberans (*n* = 10), and dedifferentiated liposarcoma (*n* = 10). The results are summarized in Table 3. Among all 50 SFT samples, STAT6 were positive in all the cases of SFT samples. 44 (88%) stained positive for TFE3 (Figure 2). TFE3 nuclear staining was strongly positive (3+) in 36 (72%) cases, moderately positive (2+) in 4 (8%) cases, and weakly positive (1+) in 3 (6%) cases, and 6 (12%) cases were completely TFE3 negative.

**Figure 3:**
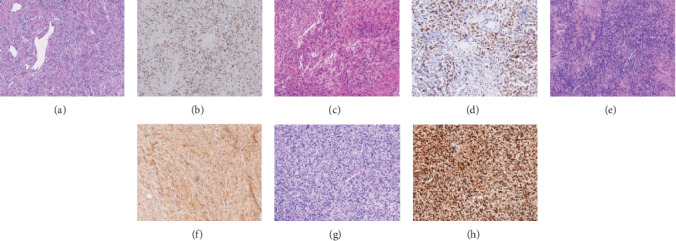
Histopathological and immunohistochemical features of SFT and other soft tumours as controls. H&E of SFT at (a) 100x and STAT6 positive expression of SFTat (b) 100x, H&E of malignant peripheral nerve sheath tumour at (c) 100x and positive Sox-10 expression in malignant peripheral nerve sheath tumour at (d) 100x, H&E staining of dermatofibrosarcoma protuberans at (e) 100x, and positive CD34 expression in dermatofibrosarcoma protuberans at (h) 100x. H&E staining of dedifferentiated liposarcoma at (g) 100x and positive MDM2 expression in dedifferentiated liposarcoma at (h) 100x.

Among cases of malignant peripheral nerve sheath tumour (*n* = 10), synovial sarcoma (*n* = 10), spindle cell lipoma (*n* = 10), dermatofibrosarcoma protuberans (*n* = 10), and dedifferentiated liposarcoma (*n* = 10) samples were used as controls. The cases of malignant peripheral nerve sheath tumour we used as controls were S-100 and Sox-10 positive, while STAT6 was negative. CD34 and STAT6 were all negative in cases of synovial sarcoma. Dermatofibrosarcoma protuberans were CD34 diffusely positive and STAT6 negative. MDM2 and CDK4 were both positive in dedifferentiated liposarcoma cases used as controls (Figure 3).

**Figure 4:**
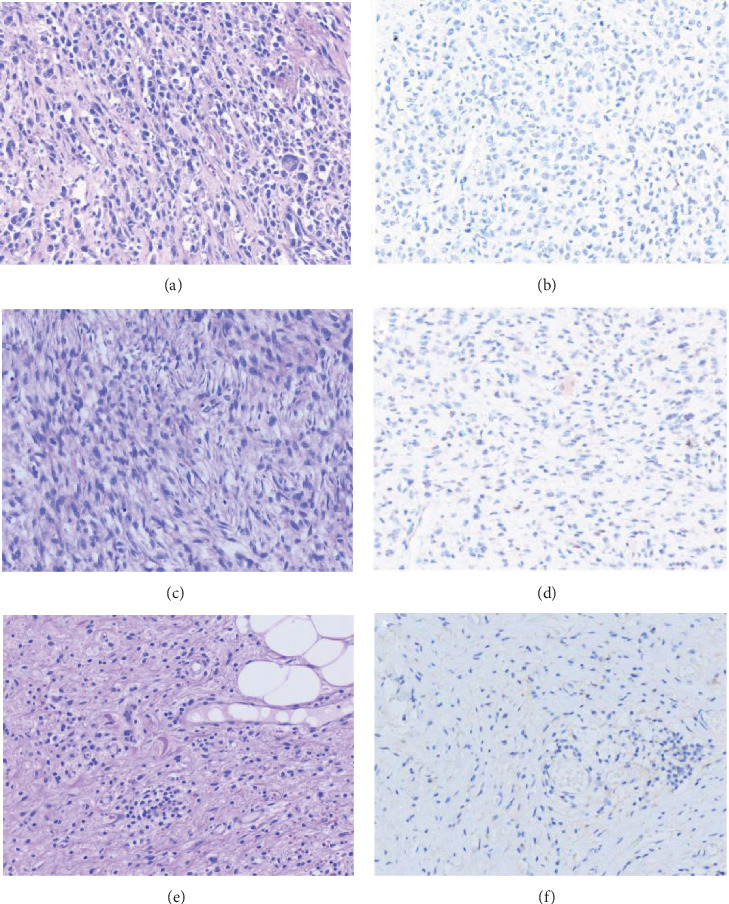
Histopathological features and TFE3 expression in other soft tumours as controls. Routine H&E of malignant peripheral nerve sheath tumour at (a) 200x, negative TFE3 expression in malignant peripheral nerve sheath tumour at (b) 200x, routine H&E staining of dedifferentiated liposarcoma at (c) 200x, negative TFE3 expression in dedifferentiated liposarcoma at (d) 200x, routine H&E of spindle cell lipoma at (e) 200x, and negative TFE3 expression in spindle cell lipoma at (f) 200x.

All examined cases of malignant peripheral nerve sheath tumour, synovial sarcoma, spindle cell lipoma, and dermatofibrosarcoma protuberans were TFE3 negative (Figure 4). Two cases (*n* = 2) of dedifferentiated liposarcoma were moderately positive for nuclear TFE3 (2+).

### 3.3. FISH Analysis

**Figure 5:**
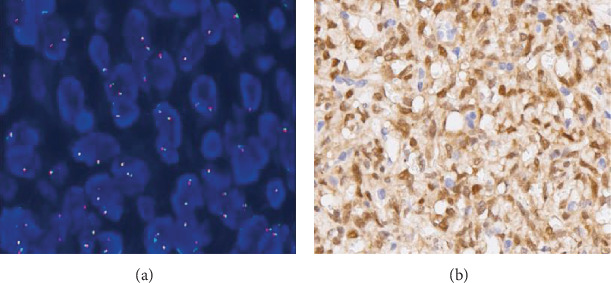
Images presenting TFE3 FISH staining. TFE3 break-apart probe assay enabled the visualization of normal fused hybridization signals (a)1000x. The SFT cases with strongly positive TFE3 at (b) 400x.

We next selected SFT samples (*n* = 10) that had shown a strong nuclear TFE3 expression previously in the IHC analysis, to conduct FISH. Using a break-apart FISH assay, we observed a combination of signals indicating a lack of split signals in all the cases, indicating a lack of the *TFE3* gene rearrangement (Figure 5).

### 3.4. Association of TFE3 Expression, Clinically Pathologic Characters, and the Outcomes

The TFE3 expression in SFTs did not correlate with clinicopathologic characteristics and outcome. There was no association of TFE3 with gender (*p* = 0.674), tumour size (*p* = 0.8486), and age (*p* = 0.5604). The mitotic rate separated the cases in two groups (≥4 and <4), and TFE3 expression was not associated with the mitotic rate (*p* = 1.0) (Table 4). On analysing event-free survival using the Kaplan-Meier analysis, the patients positive for TFE3 expression did not have a significantly poor event-free survival than the cases with no expression (*p* = 0.3425). During the follow-up, six of 44 TFE3-positive SFT patients relapsed and three patients died. No recurrence or death occurred in six SFT patients who tested negative for TFE3.

## 4. Discussion

SFT is a ubiquitous mesenchymal tumour of the fibroblastic type with a prominent haemangiopericytoma-like branching vascular pattern which often occurs in middle-aged adults and affects females and males equally. SFT shows a distinctive combination of ovoid- to spindle-shaped cells, irregular growth pattern, marked stromal collagen, and thin-walled branching haemangiopericytoma-like vessels. Nevertheless, it is often difficult to diagnose SFT and to distinguish it from other tumours of soft tissues owing to the wide histological variability, such as spindle cell lipoma, synovial sarcoma, the malignant peripheral tumour of the nerve sheath, dermatofibrosarcoma protuberans, and dedifferentiated liposarcoma. SFT and its differential diagnosis must be distinct for proper prognosis and treatment.

STAT6 and CD34 expressions were detected in SFT. There was no overexpression of p53 in SFT. The morphological characteristics between SFT and other mesenchymal spindle cell tumours are similar, such as malignant peripheral nerve sheath tumour, synovial sarcoma, spindle cell lipoma, dermatofibrosarcoma protuberans, and dedifferentiated liposarcoma. These spindle cell tumours need to be differentiated from SFT by immunohistochemical markers. Malignant peripheral nerve sheath tumours are often S-100 and Sox-10 positive. While Bcl-2 is negative, CD34 and STAT6 are negative in synovial sarcoma and can thus aid in differentiating from SFT. Dermatofibrosarcoma protuberans are often CD34 diffuse positive and STAT6 negative. Likewise, MDM2 and CDK4 are both positive in dedifferentiated liposarcoma that can thus be distinguished from SFT.

Immunohistochemical markers are vital in SFT diagnosis. While more than 90% of SFTs are CD34 positive [20], it is not specific because of its shared expression in many spindle cell tumours. For instance, CD34 is diffuse positive in dermatofibrosarcoma protuberans. TFE3 is a useful marker for distinguishing SFT from most mimics. Particularly among other CD34 positive tumours, STAT6 is a useful SFT diagnostic marker due to the NAB2-STAT6 fusion in SFTs [21] and some other soft tissue tumours, such as dedifferentiated liposarcoma express STAT6. Gene expression studies in SFTs revealed 93% of SFTs to be positive for GRIA2 while 75% DFSP was positive for GRIA2 [8]. Thus, it is difficult to identify SFT and DFSP through GRIA2.

We detected TFE3 translocation, through FISH assay, in 10 TFE3 strongly positive SFT cases. Two samples displayed weak signals for TFE3 in dedifferentiated liposarcoma among control tumours, and 88% of SFT samples showed a strong presence of TFE3 indicating that TFE3 can act as a potential marker for the diagnosis of SFT.

TFE3 is immunologically highly specific and sensitive. Diffuse and strong TFE3 staining always correlates with translocation status. The fusion or amplification of *TFE3* has been identified with molecular events such as perivascular epithelioid cell tumours (PEComas) and renal cell carcinoma associated with Xp11.2 translocation [22–24]. To evaluate whether the strong expression of TFE3 in SFT is caused by translocation, we detected TFE3 translocation, through FISH assay, in 10 TFE3 strongly positive SFT cases. Further, the results indicate that no relationship exists between nuclear TFE3 immunohistochemical staining and TFE3 translocation in SFT. The results suggest that while TFE3 was overexpressed in 88% SFC samples, the mechanism behind this increased expression is yet to be unravelled. TFE3 alterations may not be a major molecular event driving tumorigenesis in soft tissues. Studies have found that the PI3K-AKT-mTORC1 signalling pathway, the Wnt/beta-catenin signalling pathway, and the TGF-beta signalling pathway are mainly associated with TFE3 regulation. TFE3 are regulated by nutritional status. During satiety, mTORC1-dependent phosphorylation induces TFE3 to remain in the cytoplasm, while during starvation, TFE3 translocates into the nucleus and induces transcription of several genes associated with lysosomes and autophagy [25]. TFE3 activates Wnt/beta-catenin signals, forming a positive feedback loop between members of the MITF family and the Wnt signalling pathway [26, 27]. Brady et al. observed that when DNA damage occurs, p53 activates TFE3 by inhibiting mTORC1 activity. This p53-dependent TFEB and TFE3 activation further enhance the p53 signal through feedback and the negative feedback mechanism, resulting in the increase of p53 stability and protein level, leading to the increased expression of many transcription factors involved in DNA damage response and repair [28]. However, the mechanism of TFE3 overexpression in SFT is still unclear as there was no rearrangement of this gene shown by FISH. We speculate that the expression of TFE3 in SFT may be associated with autophagy and the PI3K-AKT-mTORC1 signalling pathway. However, the underlying mechanism needs more in-depth research.

The biological behaviour of SFT is still uncertain. They can turn into malignant tumours and recur at a rate of about 10%. They can also invade surrounding tissues and organs, such as the vertebral body, and compress the spinal cord, which indicates that they are low-grade or potentially malignant tumours. Malignant SFTs account for about 20% of tumours. They may be primary or benign in nature and their biological behaviour depends on the size and growth pattern of tumours. Malignant SFTs are usually hypercellular lesions, showing increased mitosis (>4 mitosis events per 10 HPF), variable cytological atypia, tumour necrosis, and/or infiltrative margins [4, 29]. Our results indicate the nonsignificance of TFE3 expression with respect to the biological behaviour of SFT. All the six TFE3-negative cases in SFTs did not have a relapse, and all the patients were in good condition on follow-up in this study. Our results also show that TFE3 expression has no relationship with the age, tumour size, gender, and mitosis events of SFT patients.

## 5. Conclusions

In SFT, TFE3 immunohistochemical staining is a useful diagnostic marker and can be combined with STAT6 for better diagnosis, as very few soft tissue tumours are TFE3 positive. We also verified that the increase in TFE3 expression was unrelated to Xp11 gene translocation events. Our findings offer novel insights into the diagnosis, aetiology, and molecular mechanisms of SFT.

## Figures and Tables

**Table 1:** Panel of antibodies used in this study.

Antigen	Clone	Dilution	Source
CK	AE1/AE3	Prediluted	Dako
Vimentin	V9	Prediluted	Dako
S-100	Polyclonal	Prediluted	Dako
Sox-10	ZA-0624	Prediluted	ZSGB-BIO
CDK4	ZA-0614	Prediluted	ZSGB-BIO
MDM2	ZA-0425	Prediluted	ZSGB-BIO
CD34	QBEND 10	Prediluted	Dako
STAT6	ZA-0647	Prediluted	ZSGB-BIO
TFE3	ZA-0657	Prediluted	ZSGB-BIO
Ki-67	MIB-1	Prediluted	Dako

**Table 2:** Patient clinicopathologic data.

	SFT (*n* = 50)	MPNST (*n* = 10)	DFSP (*n* = 10)	SCL (*n* = 10)	SS (*n* = 10)	DDLPS (*n* = 10)
Age (y)	18-82	23-75	19-47	45-63	47-72	43-76
Mean (y)	49.8	51.2	34.4	54	55.7	61.9
Male	21	4	2	5	8	7
Female	29	6	8	5	2	3
Location						
Retroperitoneum	14	2	0	0	0	10
Pleura	4	0	0	0	0	0
Back	0	2	2	2	2	0
Kidney	2	1	0	0	0	0
Buttock	3	1	0	2	2	0
Limb	2	2	4	2	6	0
Others	25	2	4	4	0	0

SFT: solitary fibrous tumour; MPNST: malignant peripheral neurilemmoma; DFSP: dermatofibrosarcoma protuberans; SCL: spindle cell lipoma; SS: synovial sarcoma; DDLPS: dedifferentiated liposarcoma.

**Table 3:** TFE3 immunohistochemical staining results.

	*n*	0	1+	2+	3+	Total (%)
SFT	50	6	3	4	36	88
MPNST	10	10	0	0	0	0
DFSP	10	10	0	0	0	0
SCL	10	10	0	0	0	0
SS	10	10	0	0	0	0
DDLPS	10	8	0	2	0	20

SFT: solitary fibrous tumour; MPNST: malignant peripheral nerve sheath tumour; DFSP: dermatofibrosarcoma protuberans; SCL: spindle cell lipoma; SS: synovial sarcoma; DDLPS: dedifferentiated liposarcoma; TFE3: transcription factor E3.

**Table 4:** Association of TFE3 expression with clinicopathological characteristics in 50 patients with solitary fibrous tumour.

	All patients (*n* = 50)	TFE3 negative	TFE3 positive	*p* value
Gender				
Female	27	4	23	*p* = 0.6740
Male	23	2	21	
Ages (years), mean ± SD	52.1 ± 2.4	48.3 ± 6.6	52.6 ± 2.6	*p* = 0.5604
Tumour size (cm), mean ± SD	7.45 ± 0.7	7.4 ± 0.8	7.8 ± 1.5	*p* = 0.8486
Mitotic rate				
≥4	10	1	9	*p* = 1.0
<4	40	5	35	

SD: standard deviation.

## Data Availability

The data used to support the findings of this study are available from the corresponding author upon request.
